# Special Issue “New Agents and Novel Drugs Use for the Oncological Diseases Treatment”

**DOI:** 10.3390/ijms27031454

**Published:** 2026-02-01

**Authors:** Alexandra Zakharenko, Nadezhda Dyrkheeva

**Affiliations:** Institute of Chemical Biology and Fundamental Medicine, Siberian Branch of the Russian Academy of Sciences, 630090 Novosibirsk, Russia

Cancer is the second leading cause of death in the world after cardiovascular disease. Despite certain advances in diagnosis and treatment methods, the prevalence of cancer among the population is growing every year, and this trend does not depend on the economic status of the country or social status. Therefore, the development of new antitumor agents remains an important task.

Exploratory research is being conducted in many laboratories around the world, and both synthetic and natural compounds or their derivatives with anticancer properties are being investigated. Non-primary use of known drugs is also under research.

This Special Issue contains original articles and reviews addressing the search for new chemotherapeutic agents, approaches and the repurposing of known drugs for the treatment of oncological diseases.

Glioblastoma (GB) is one of the most malignant types of tumors, with a very short survival rate (15 months) and a poor prognosis. The standard treatment for patients with glioblastoma is surgery followed by adjuvant radiation and chemotherapy, which often fail to produce the desired effect, significantly increase the cost of treatment, and are associated with complications [1]. Any expansion of treatment options and the search for new approaches will be a significant step toward improving the prognosis and life expectancy of patients. One review and five research articles in the Special Issue are devoted to this topic.

The review “Combination of Oncolytic Virotherapy with Different Antitumor Approaches against Glioblastoma” considers a new treatment option for GBM: the use of oncolytic viruses (Contribution 1). The review considers different approaches to the treatment of GB using oncolytic viruses (Selective viral replication in tumor cells; direct cell lysis followed by cascade infection of neighboring tumor cells; induction of tumor proinflammatory microenvironment, turning immunologically “cold” tumors into “hot” ones; enhancing the oncotoxic ability of oncolytic viruses through the insertion of therapeutic immune-modulators genes; viral infection of tumor-associated cells like endotheliocytes and breakdown of tumor vessels). Also discussed are the types (families) of oncolytic viruses, variants of transgenes that can both modulate the immune response against the tumor and induce or enhance tumor cell death, and combinations of oncolytic viruses with other approaches to the treatment of GB: surgery, chemotherapy, radiotherapy, and immunotherapy.

The articles published in the Special Issue proposed small molecules that inhibit essential cellular processes in GB cells (Contributions 2, 3) and improve the efficacy of alkylating anti-tumor agent temozolomide (Contribution 4), also an anti-proliferative aptamer (Contribution 5) and single-domain antibody, specific to EGFRvIII, the prevalent EGFR mutation found in GB cells (Contribution 6).

The topic of converting cold tumors into hot ones, touched upon in the review by Ageenko et al. (Contribution 1), is discussed in more detail in the review «Breast Cancer Treatment Strategies Targeting the Tumor Microenvironment: How to Convert “Cold” Tumors to “Hot” Tumors» (Contribution 7). The role of the tumor microenvironment (TME) in tumor immunogenicity is analyzed, and strategies for editing the tumor microenvironment for breast cancer treatment using nanoparticles are considered.

The review by Feng et al. (Contribution 8) examines the conceptual classifications and benefits of immunotherapy, as well as the use of new drugs and various immunotherapeutic agents and methods with different mechanisms for the treatment of cancer.

The review by De et al. (Contribution 9) examines the potential of ultrasmall nanoparticles (USNPs) for drug delivery, discussing their physicochemical properties, structural diversity, applications in the treatment of cancer and neurological diseases, vaccine delivery, and imaging. Unresolved issues associated with the use of USNPs and potential research directions are also discussed.

The topic of nanomaterials is also addressed in several articles in the Special Issue. For example, Manmuan et al. (Contribution 10) describe the creation of D-Limonene Nanoemulsions for the suppression of oral cancer using Ostwald ripening inhibitors. The most effective particle composition was identified, and their physical properties and effects on the oral cancer cell line (KON), suppressing cell viability, metastasis, and invasion, are described. The molecular mechanisms of this effect are demonstrated. Alotaibi et al. (Contribution 11) describe the creation and characterization of a Zeolitic Imidazole Framework/Silica Nanocomposite with platinum complexes, demonstrating their cytotoxic/antiproliferative effects on tumor cells.

The importance of derivatives of natural compounds in the development of antitumor agents deserves special mention. Among the antitumor agents approved for clinical use since the 1980s, a third are natural compounds or their derivatives [2]. This Special Issue publishes a series of studies based on these compounds.

Kornienko et al. (Contribution 12) propose using the usnic acid derivative, the Tdp1 inhibitor AF-185, as an additional component of tumor therapy in combination with topotecan. The study demonstrated that the Tdp1 inhibitor not only sensitizes mice Krebs-2 and Lewis carcinomas to the action of topotecan, but also normalizes the state of the peripheral blood of mice, which is disturbed in the presence of a tumor. The effect of another usnic acid derivative, the Tdp1 inhibitor OL9-119, on the cell transcriptome is the subject of an article by Zakharenko et al. (Contribution 13). OL9-119, capable of potentiating the antitumor effect of topotecan, was found to be able to reduce cell motility by decreasing the expression of a number of genes, which may explain the antimetastatic effect of this compound. Warburgia salutaris extracts also exhibited antiproliferative and antimetastatic effects on MCF-7 breast cancer cells by promoting apoptosis (Contribution 14). Clerodendrum chinense leaf extract (CCL) effectively suppressed colony formation and cell migration in a dose-dependent manner in MCF-7 breast cancer and HeLa cervical cancer cells (Contribution 15). The composition of CCL and its molecular mechanism of action have been studied. O-alkyl derivatives of secondary metabolites of plants E-chalcones exhibit potent activity against breast cancer MDA-MB-231 and MCF-7 cells, as well as colorectal cancer HCT-116 and cervical cancer HeLa cell lines (Contribution 16). These compounds also inhibit EGFR and VEGFR-2, proteins that play a key role in cancer development, and possess notable anti-estrogenic activity, which may offer dual therapeutic benefits in the treatment of hormone-dependent cancers.

Efendiev et al. (Contribution 17) report the first combined use of photodynamic therapy and chemotherapy with selective intra-arterial administration of a photosensitizer and a chemotherapeutic drug for the treatment of cancer in humans. Both patients of the pilot study demonstrated significant tumor volume reduction and concomitant tissue regeneration following combination therapy, which enabled the patients to transition to an operable condition without significant functional or cosmetic defects, providing the possibility of further radical surgical treatment that preserves anatomical integrity and physiological function.

Cancer cells use metabolic reprogramming and epigenetic modifications to support their abnormal proliferation. Maksimova et al. (Contribution 18) briefly review the role of epigenetic mechanisms in carcinogenesis and tumor progression. The mechanism of action of Curaxin CBL0137, which is capable of activating epigenetically silenced genes, is studied. The study demonstrates the potential of CBL0137 for the treatment of tumors with aberrant epigenetic profiles (DNMT and BET overexpression). The possibility of gene expression reprogramming in HepG2 hepatocarcinoma cells using citrate, a key metabolite of the tricarboxylic acid cycle, was examined in (Contribution 19). Citrate was used to detect more than 3500 differentially expressed genes and approximately 40 altered signaling pathways, including cytochromes, which could both improve the effectiveness of chemotherapeutics and reduce the aggressiveness of tumors by diminishing cell migration and invasion. Another attempt to interfere with tumor cell metabolism is described in (Contribution 20): unlike normal cells, leukemia cells are incapable of synthesizing asparagine on their own, and the depletion of extracellular L-asparagine through L-asparaginase (ASNase) administration leads to disruption of protein synthesis and leukemia cell death. The article is devoted to the development of a test system for assessing the activity of different L-ASNases in biological environments, taking into account potential interfering factors and matrix effects, as well as ASPNase formulations (conjugates with polyamines or polyelectrolyte complexes).

Imipridone derivative TR-57, an inhibitor of mitochondrial caseinolytic protease ClpXP, causes the fragmentation of mitochondria and an essential decrease in the number of mitochondrial nucleoids/mtDNA in SUM159 human breast cancer cells (Contribution 21). Long-term incubation with TR-57 leads to decreased Complexes I–IV protein levels and the complete suppression of mitochondrial respiration without complete loss of mitochondrial membrane potential.

A very interesting study by Adornetto et al. (Contribution 22) describes the off-label use of the antidepressant amitriptyline to suppress human SH-SY5Y neuroblastoma cells. Treatment with amitriptyline resulted in reduced cell viability and decreased clonogenic capacity, as well as modulation of autophagy. This study provides groundwork for further research into the use of amitriptyline in oncotherapy and also highlights the need for research into the effects of long-term therapy with this drug.

Adriaanse et al. (Contribution 23) propose the use of combination therapy with menin and DOT1L inhibitors, key proteins in KMT2A-rearranged leukemogenesis. KMT2A-rearranged pediatric acute myeloid leukemia (AML) and acute lymphoblastic leukemia (ALL) develop or are initially resistant to the menin inhibitor revumenib; the study demonstrates synergy between revumenib and the DOT1L inhibitor pinometostat in KMT2A-rearranged ALL, as well as different responses of KMT2A-rearranged ALL and AML cells to revumenib treatment, suggesting precise patient stratification to optimize the use of menin inhibitors.

The topic of overcoming the resistance of childhood cancer tumor cells is continued in the work by Wilke et al. (Contribution 24): the compound (2,6-dimethylphenyl)arsonic acid (As2) demonstrated significant inhibition of cell proliferation and induction of apoptosis in leukemia and lymphoma cells while sparing healthy leukocytes; overcomes multidrug resistance and sensitizes drug-resistant leukemia and lymphoma cell lines to treatments with the common cytostatic drugs.

The articles and reviews published in this Special Issue cover various approaches and rationales for the search for new agents and treatments for oncological diseases. The importance of finding treatments for glioblastoma, one of the most severe diagnoses with a poor prognosis, is emphasized. Also noteworthy are the growing popularity of antitumor agents among natural compounds and their derivatives, the active development of nanomaterials with improved pharmacological properties, research into the possibility of editing the tumor microenvironment and metabolism, and other possibilities.
